# Epidemiological and Clinical Characteristics of Mpox in Cisgender and Transgender Women and Non-Binary Individuals Assigned to the Female Sex at Birth: A Comprehensive, Critical Global Perspective

**DOI:** 10.3390/v16030325

**Published:** 2024-02-21

**Authors:** Nicola Luigi Bragazzi, Woldegebriel Assefa Woldegerima, Jianhong Wu, Manlio Converti, Lukasz Szarpak, Andrea Crapanzano, Marwan Odeh, Raymond Farah, Rola Khamisy-Farah

**Affiliations:** 1 Laboratory for Industrial and Applied Mathematics (LIAM), Department of Mathematics and Statistics, York University, Toronto, ON M3J 1P3, Canada; wassefaw@yorku.ca (W.A.W.); wujhhida@gmail.com (J.W.); 2 Postgraduate School of Public Health, Department of Health Sciences (DISSAL), University of Genoa, 16126 Genoa, Italy; 3 United Nations Educational, Scientific and Cultural Organization (UNESCO), Health Anthropology Biosphere and Healing Systems, University of Genoa, 16126 Genoa, Italy; 4 Department of Food and Drugs, University of Parma, 43125 Parma, Italy; 5 ASL Napoli 2 Nord, 80027 Naples, Italy; manlioconverti@gmail.com; 6 Department of Clinical Research and Development, LUXMED Group, 85-871 Warsaw, Poland; lukasz.szarpak@gmail.com; 7 Colorectal Cancer Unit, Maria Sklodowska-Curie Bialystok Oncology Center, 15-027 Bialystok, Poland; 8 Henry JN Taub Department of Emergency Medicine, Baylor College of Medicine, Houston, TX 77030, USA; 9 Department of Counseling, San Francisco State University, San Francisco, CA 94132, USA; andreacrapanzanophd@gmail.com; 10 Department of Obstetrics and Gynecology, Galilee Medical Center, Nahariya 2210001, Israel; marwan20@bezeqint.net; 11 Azrieli Faculty of Medicine, Bar-Ilan University, Safed 1311502, Israel; raymond.f@ziv.health.gov.il (R.F.); rkhamisy@yahoo.com (R.K.-F.); 12 Department of Medicine B, Ziv Medical Center, Safed 13100, Israel; 13 Clalit Health Service, Akko 2412001, Israel

**Keywords:** Mpox, cisgender, transgender women, non-binary individuals assigned to female sex at birth, gender-specific differences, gender medicine

## Abstract

The 2022–2023 Mpox multi-country outbreak, identified in over 110 WHO Member States, revealed a predominant impact on cisgender men, particularly those engaging in sex with men, while less frequently affecting women. This disparity prompted a focused investigation into the gender-specific characteristics of Mpox infections, particularly among women, to address a notable knowledge gap. This review systematically gathers and analyzes the scientific literature and case reports concerning Mpox infections in women, covering a broad geographical spectrum including regions such as Argentina, Brazil, Colombia, Nigeria, Europe, Vietnam, and the United States. The analysis delves into various aspects of Mpox in women, including clinical features, epidemiology, psychological impacts, preparedness strategies, and case studies, with particular attention to pregnant women and those with underlying health conditions. Empirical data from multiple studies underscore the unique epidemiological and clinical patterns of Mpox in women. In the United States, a small percentage of Mpox cases were reported among cisgender women, with a notable portion involving non-Hispanic Black or African American, non-Hispanic White, and Hispanic or Latino ethnicities. The primary transmission route was identified as sexual or close intimate contact, with the virus predominantly manifesting on the legs, arms, and genital areas. Further, a study in Spain highlighted significant disparities in diagnosis delays, transmission modes, and clinical manifestations between genders, indicating a different risk profile and disease progression in women. Additionally, a case from Vietnam, linked to a new Mpox sub-lineage in women, emphasized the role of women in the transmission dynamics and the importance of genomic monitoring. This review emphasizes the necessity for inclusive surveillance and research to fully understand Mpox dynamics across diverse population groups, including women. Highlighting gender and sexual orientation in public health responses is crucial for an effective approach to managing the spread and impact of this disease. The findings advocate for a gender-diverse assessment in health services and further research to explore the nuances of Mpox transmission, behavior, and progression among different groups, thereby enhancing the global response to Mpox and similar public health challenges.

## 1. Introduction

Mpox (formerly known as Monkeypox) is a zoonotic disease caused by a double-stranded DNA virus from the Orthopoxvirus *genus*, belonging to the same family of viruses that cause smallpox and smallpox-like infections (*Poxviridae*) [1]. This disease was first identified in humans in 1970 in the Democratic Republic of the Congo (DRC) in a region where smallpox had been eliminated in 1968 and had been declared as successfully eradicated in the whole country in 1971, after the completion of a mass vaccination campaign, conducted amidst dramatic challenges, including extreme poverty, a weak, deteriorating health system, and civil strife [2].

Since then, Mpox has been reported in humans in several other central and western African countries [3,4]. Characterized by flu-like symptoms, such as a fever, fatigue, lymphadenopathy, and a rash, it generally spans 2–4 weeks, often resolving itself, though severe cases can occur in immunocompromised individuals [5]. A distinctive feature of Mpox is the rash that develops, often beginning on the face and then spreading to other parts of the body, including the palms of the hands and the soles of the feet. The rash goes through different stages, including macules, papules, vesicles, pustules, and crusts, before healing [6]. Mpox is primarily transmitted to humans through close contact with infected animals, such as rodents and primates, or their bodily fluids. Human-to-human transmission can occur through close physical contact with infected individuals or their contaminated materials, such as bedding or clothing. Respiratory droplets can also spread the virus, but this requires prolonged face-to-face interactions [7,8,9].

In early April–May 2022, Mpox cases were detected and described also in countries without direct or immediate epidemiological links to West or Central Africa. Given the emerging epidemiological and clinical features of the cases, on 23 June 2022, the “World Health Organization” (WHO) declared Mpox an evolving threat of moderate public health concern [10] and then, on 23 July 2022, a “Public Health Emergency of International Concern” (PHEIC) [11,12].

In countries within Africa where Mpox is endemic, this disease is transmitted through two closely related genetic groups: Clade I (previously known as Congo Basin) and Clade IIa (previously known as West Africa). Both types can lead to infections that pose a serious risk to life, even if to varying degrees. However, the molecular signature of the 2022–2023 Mpox multi-country outbreak appears to significantly differ [13,14]. Also, from an epidemiological standpoint, during this outbreak, the majority of cases were observed in cisgender men having sex with men (cMSM), with women being less frequently affected [15,16,17].

According to the available data related to the 2022–2023 Mpox multi-country outbreak, 3141 out of the 87,036 confirmed Mpox cases (3.6%) involved women, mostly from the WHO Region of the Americas (2336 out of 3141, 74%) and who were exposed to the virus via sexual encounters (260 out of 507 cases for which the transmission route was documented, 51%) [18]. However, to the best of our knowledge, the sex- and gender-specific characteristics of Mpox infections in terms of differential epidemiological trends, impacts, and clinical features have not been comprehensively appraised, despite the importance and necessity of integrating sex and gender considerations into emerging infectious disease management to mitigate the magnification of existing inequities and violations of principles of fairness and human rights [19].

Therefore, to fill in this gap of knowledge, we systematically collected all references to scientific articles, including clinical reports, case series, and cohort studies, concerning Mpox infections, particularly focusing on the female population, encompassing cases from specific populations, like pregnant or breastfeeding women, female sex workers, or female patients with underlying comorbidities, from various geographical locations. The present review covers various aspects such as distinctive clinical characteristics and epidemiological features that warrant enhanced surveillance and tailored management policies. We also discuss the infection’s psychological impacts on women, country-specific preparedness strategies, and implications for female sexual, reproductive, and overall health [20,21], as well as the importance of global initiatives addressing Mpox in particular, generally overlooked groups, such as pregnant women [22,23,24], and gender-diverse individuals, including transgender women [25].

## 2. Materials and Methods

### 2.1. Study Protocol and Ethical Considerations

Before commencing the literature search, an a priori study protocol was drafted, consulting an expert librarian, in accordance with the “Preferred reporting items for systematic review and meta-analysis protocols” (PRISMA-P) checklist [26]. A multidisciplinary team was established, consisting of experts in research methodology (N.L.B. and L.S.), the mathematical modeling of communicable diseases (W.A.W. and J.W.), queer/LGBT-medicine (M.C. and A.C.), internal medicine and infectious diseases (R.F.), gynecology and obstetrics (M.O. and R.K.-F.), and gender medicine (R.K.-F.). This study was designed to ensure inclusivity and the consideration for diversity within gender categories, particularly emphasizing the inclusion of transgender and non-binary individuals.

### 2.2. Study Aims and Objectives

The main objective was to delve into the clinical impact in terms of symptoms and clinical progressions of Mpox in women and compare these findings to those observed in men, paying special attention to any differences in severity, presentation, and potential complications. An in-depth look into the epidemiology of Mpox among women was also undertaken to understand incidence/prevalence rates, identify risk factors, and explore any disparities in access to care or outcomes that may exist. An important aspect of this study involved analyzing the predominant routes of Mpox transmission among women and contrasting these with the trends observed in men, highlighting any gender-specific behaviors or trends that may influence transmission dynamics. Moreover, this study aimed to evaluate the effectiveness and outcomes of Mpox treatments in women compared to men, taking into account aspects such as side effects, recovery durations, and the necessity for gender-specific treatment adaptations.

### 2.3. Search Strategy

To conduct a systematic literature review focusing on the impact of Mpox on women, we employed a comprehensive search strategy using specific keywords to ensure the inclusion of relevant studies. The search was performed across multiple electronic scholarly databases, including MEDLINE via its publicly accessible interface PubMed, Scopus, Web of Science, and EMBASE, to capture a wide range of scientific literature. The keywords used for the search were a combination of terms related to this disease (“monkeypox” OR “Mpox”) and those specifying the population of interest by including them in the title/abstract of the articles (“female”, “females”, “woman”, “women”, “transwoman”, “transwomen”, “male-to-female”, “lactation”, “pregnant”, “pregnancy”, and “breastfeeding”). This approach was designed to specifically target studies that focus on women or females, thus allowing for a focused review of the gender-specific aspects of Mpox infections. We embraced an expanded, inclusive definition of women, incorporating transgender women and non-binary individuals assigned to the female sex at birth. The search was supplemented by manual searches of the reference lists of identified articles and of target journals to ensure comprehensive coverage.

### 2.4. Inclusion and Exclusion Criteria

Inclusion/exclusion criteria were devised according to the “Population/patients—Exposure—Comparator/comparison—Outcome(s)—Study design” (PECOS) mnemonic. In studying the impact of Mpox on different genders, the focus was on women affected by the virus, including cisgender and transgender women, as well as non-binary individuals assigned female at birth (P, population/patients). Exposure (E) to the Mpox virus had to be laboratory-confirmed. The examination extends to comparing the experiences of women with those of men, encompassing cisgender, transgender, and non-binary individuals assigned male at birth (C, comparator/comparison). The key areas of investigation include the clinical manifestations of Mpox, its epidemiological features, the routes through which it is transmitted, and the responses to various treatment options (O, outcomes). In terms of the study design (S), eligible studies included peer-reviewed articles, case reports, case series, and observational studies that provided data on clinical characteristics, epidemiological features, and outcomes of Mpox infections in women.

### 2.5. Selection and Identification Process of Eligible Studies

The selection process involved screening titles and abstracts for relevance, followed by a thorough full-text review to confirm eligibility, ensuring a methodical and exhaustive review of the literature on the subject.

Further details are reported in Table 1.

### 2.6. Data Synthesis and Finding Reporting

The data were synthesized, and the findings were reported in accordance with the “Preferred reporting items for systematic review and meta-analysis” (PRISMA) checklist [27].

### 2.7. Gray Literature

Besides the peer-reviewed literature, some major institutional websites of national/international public health authorities and organisms were searched, including the sites of the WHO, the “Public Health Agency of Canada” (PHAC), the USA “Centers for Disease Control and Prevention” (CDC), the “European Centre for Disease Prevention and Control” (ECDC), and the “UK Health Security Agency” (UKHSA).

### 2.8. Expected Study Outcomes

The expected outcomes include gaining nuanced insights into the gender-specific impacts of Mpox, which can inform the development of tailored public health strategies and clinical guidelines. By enhancing the understanding of Mpox’s effects on women, particularly those from marginalized gender identities, this research aims to contribute significantly to the global response to this disease, ensuring that interventions and public health measures are responsive to the specific needs and risks faced by women.

## 3. Results

### 3.1. Literature Search

**Figure 1:**
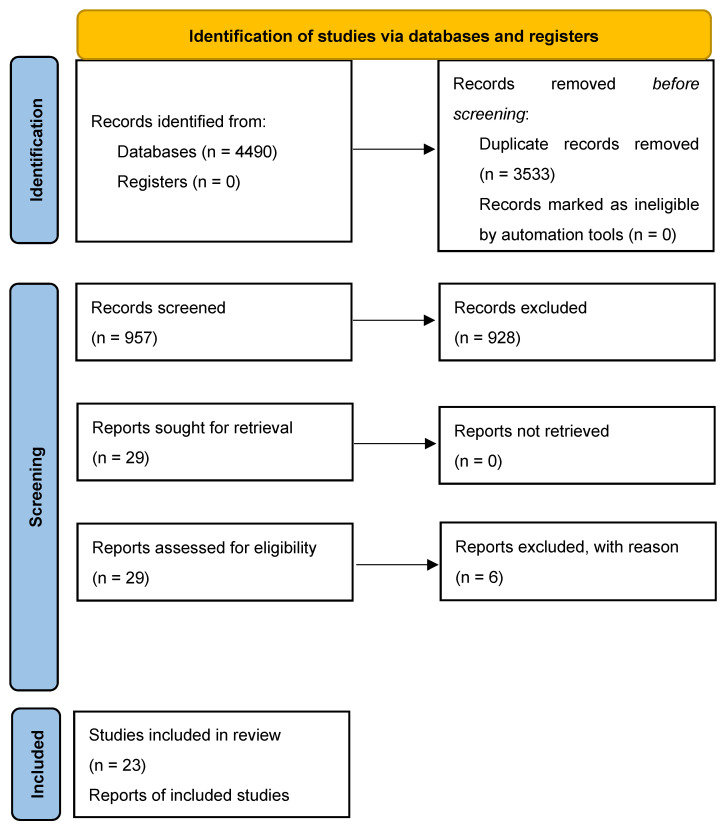
A PRISMA 2020 flow diagram depicting the search strategy adopted in the present systematic review.

The initial literature search yielded a pool of 4490 items; 3533 items were removed, being duplicates. Out of the 957 unique items, 928 were discarded, based on the title and/or abstract screening. Out of the twenty-nine studies screened for their full text, six studies were excluded with reason, while the remaining twenty-three studies [28,29,30,31,32,33,34,35,36,37,38,39,40,41,42,43,44,45,46,47,48,49,50] from various locations, including Argentina, Brazil, Colombia, Nigeria, Europe, Vietnam, and the USA, were retained and overviewed. More in detail, six cohort studies [28,29,30,31,32,33] were retrieved and synthesized, along with fourteen clinical case reports [34,35,36,37,38,39,40,41,42,43,44,45,46,47], two case series [48,49], and one case series review [50] (Figure 1). The major features of these studies are presented in Table 2.

### 3.2. Cohort Studies

In the USA, Oakley et al. [28] gathered all cases reported between 11 May and 7 November 2022 by the CDC and health departments. A total of 769 Mpox cases affected cisgender women aged 15 and older, making up 2.7% of all cases reported during this period. Based on the data collected, the median age was 32 years (interquartile range: 25–40 years; range: 15–89 years), and a significant portion of these cases involved cisgender women of non-Hispanic Black or African American (44%), non-Hispanic White (25%), and Hispanic or Latino ethnicity (23%). Most of these women (71%) reported sexual activity or close intimate contact as their likely exposure to Mpox. More specifically, the majority had recent sexual contact with cisgender men and a smaller number with cisgender women. From a clinical standpoint, the virus manifested with symptoms including a rash, headaches, pruritis, malaise, a fever, and chills. The rash was mainly located on the legs, arms, genital areas, and trunk. The distribution of rash locations was consistent regardless of whether recent sexual exposure was reported. Among those with available data on immunocompromising conditions, 9% reported having such a condition other than HIV. Among the subset with a known HIV status, 8% were HIV-positive, none of whom were pregnant. Of note, there were 23 cases (3%) of Mpox among pregnant (n = 21) or recently pregnant individuals (n = 2, within 3 weeks postpartum), all of whom were identified as cisgender women. Among those with known exposure data, sexual contact was the most reported mode of transmission, followed by household contact. The cases were fairly evenly distributed across all trimesters of the pregnancy. A rash was a universal symptom, and genital lesions were reported in some cases. However, there were no reports of genital lesions at the time of delivery. Out of the 23 cases, four required hospitalizations due to symptoms, but none required intensive care or unplanned delivery, and eleven were treated with tecovirimat without any reported adverse effects. Three main types of outcomes were reported: two full-term deliveries without complications and one spontaneous abortion. Two newborns developed lesions shortly after birth but responded well to treatment with tecovirimat, and one also received intravenous vaccinia immune globulin. There was a case of a breastfeeding individual developing lesions postpartum, with the newborn also showing symptoms later. Two other breastfeeding women diagnosed with Mpox had no transmission through breast milk, confirmed by negative PCR tests for Mpox virus DNA, underscoring the importance of monitoring and managing Mpox cases in pregnant and recently pregnant individuals, considering the potential risks to both the mother and the newborn. The effective response to newborn infections and the absence of adverse events from tecovirimat treatment are particularly noteworthy.

In Argentina, Sánchez Doncell et al. [29] conducted a study specifically focusing on women exposed to Mpox and recruited from June 2022 to February 2023, exploring Mpox’s epidemiology, clinical manifestations, and post-infection complications. Utilizing a retrospective analysis at a Febrile Emergency Unit, based in Buenos Aires, the authors examined RT-PCR-confirmed cases among women, investigating sexual health impacts. Of the 214 positive cases from 340 consultations, only 3 were female (two cisgender women and one transgender woman). Details are provided by the authors only for the two cisgender women, who were aged 31 years, one with an obstetric history of pregnancy and childbirth, both apparently healthy and immunocompetent and with a negative serology report for HIV, syphilis, or hepatitis B and C. Concerning contraception, one denied current use, and the other reported previous tubal ligation. Both were heterosexual, one with a partner positive for Mpox. One patient reported headaches, myalgias, and asthenia, while the other denied headaches and muscle aches, describing complaints of weakness, fevers, perianal pain, and lymphadenopathy. Lesions were located in the upper and lower limbs, back, and abdomen in the first case, while in the second case, they affected the upper and lower limbs, abdomen, perianal area, and face. Both denied allergies, diseases, and surgeries, reporting sexual relations in the last 21 days. No complications were reported in either case.

In Brazil, Coutinho et al. [30] obtained surveillance data of Mpox cases notified to the Rio de Janeiro State Health Department in the period from 12 June to 15 December 2022, and compared women (cisgender or transgender) to men (cisgender or transgender) using chi-squared, Fisher’s exact, and Mood’s median tests. A total of 1306 Mpox cases were reported; 1188 (91.0%) men (99.8% cisgender and 0.2% transgender), 108 (8.3%) women (87.0% cisgender and 13.0% transgender), and 10 (0.8%) non-binary persons. Compared to men, women were more frequently older (concerning the category of 40 years and older: 34.3% vs. 25.1%; *p* < 0.001), reported more frequent non-sexual contact with a potential Mpox case (21.4% vs. 9.8%; *p* = 0.004); fewer sexual partners (10.9 vs. 54.8%; *p* < 0.001); less sexual contact with a potential Mpox case (18.5% vs. 43.0%; *p* < 0.001); fewer genital lesions (31.8% vs. 57.9%; *p* < 0.001); fewer systemic Mpox signs/symptoms (38.0% vs. 50.1%; *p* = 0.015); and had a lower HIV prevalence rate (8.3% vs. 46.3%; *p* < 0.001), with all cases being among transgender women. Eight women, aged 13–69 years, were hospitalized (with a median hospitalization time of five days and an interquartile range of 3.5–7 days), with the frequency of skin rashes and hospital admissions being similar across genders. However, no deaths occurred among women, with all reported Mpox fatalities (totaling 5) being among men. In terms of epidemiological temporal trends, the highest number of cases among women was notified in epidemiological week 34, when the number of cases among men started to decrease. Specifically concerning transgender women and non-binary individuals assigned to the female sex at birth, the majority of Mpox cases among transgender women (14 cases) and non-binary individuals (10 cases) were observed in those aged 25–29 years or older, with 12 out of 14 and 9 out of 10 cases, respectively, falling into this age group. The predominant racial self-identification was Pardo for transgender women (10 out of 14) and non-binary individuals (3 out of 10), with Black being the next most common (2 out of 14 for transgender women and 3 out of 10 for non-binary individuals). Half of the individuals in each group had completed secondary education (9 out of 13 transgender women and 5 out of 10 non-binary individuals). Approximately half of both groups reported having sexual relationships exclusively with men (7 out of 8 transgender women and 4 out of 7 non-binary individuals). The majority had engaged in sexual activities with someone who could potentially have Mpox (10 out of 14 transgender women and 5 out of 6 non-binary individuals). All HIV cases among the women in this study were found in transgender women (8 out of 14), while non-binary individuals accounted for three HIV cases. There were no hospitalizations recorded for either transgender women or non-binary individuals.

In Europe, an online survey was conducted under the VACCELERATE Consortium [31], focusing on the evaluation and confirmation of Mpox cases among women across countries. The survey revealed that Spain and Belgium had the highest numbers evaluated, with Spain reporting 226 cases and Belgium 60 cases. Among those evaluated, women residing in Spain and Portugal showed the highest likelihood of infection, with ratios of 0.08 and 0.06, respectively.

Specifically concerning Spain, analyzing surveillance data, Vallejo-Plaza et al. [32] found similar temporal trends but noted disparities in diagnosis delays, sexual transmissions, and clinical manifestations between genders. In terms of prevalence and age distribution, women constituted a small fraction (2.1%) of the total Mpox cases reported in Spain during the study period, with a younger median age compared to men. Concerning the transmission mechanisms, the primary mode of transmission was close contact during sexual relations for both men and women, though a significant proportion of women had different transmission routes compared to men. Regarding the HIV infection rates, a notable disparity was observed in HIV infection rates between men and women with Mpox, suggesting differing risk profiles. In terms of symptomatology, women exhibited certain signs and symptoms at different rates than men, such as less frequent anogenital rashes but more frequent rashes in other locations. As far as diagnosis and complications were concerned, women experienced a longer delay from symptom onset to diagnosis and had higher complication rates compared to men, although no deaths were reported among women.

Globally, Thornhill et al. [33] collected data on 136 cisgender and transgender women and non-binary individuals assigned to the female sex at birth and diagnosed with the Mpox virus from 11 May to 4 October 2022, across 15 countries. The median age was 34 years, with a range from 19 to 84 years. The group included 62 transwomen, 69 cis-women, and five non-binary individuals, with the latter two categories combined for analysis. In terms of sexual orientation, 108/136 (79%) were heterosexual, while 10/136 (7%), 2/136 (1%), and 16/136 (12%) were bisexual, lesbian, and unknown, respectively. A significant majority, consisting of 121 participants, had sexual contact with men. HIV prevalence was notable, especially among transwomen (50% of transwomen compared to 8% of cis-women and non-binary individuals). The majority of transwomen (89%) and a lesser proportion of cisgender women and non-binary individuals assigned female at birth (61%) were suspected of contracting the virus through sexual contact, while cisgender women and non-binary individuals assigned female at birth also reported non-sexual transmission routes. Misdiagnosis occurred in 34% of the cisgender women and non-binary individuals assigned female at birth. The data show that 93% had a rash, predominantly anogenital (74%) and vesiculopustular (87%). Lesions were common, with a median count of ten. Over half of the participants had mucosal lesions, which were correlated with vaginal and anal sexual activities. PCR tests confirmed Mpox virus DNA in all vaginal swabs taken. Hospitalization was necessary for 13% of the cases, mainly for bacterial superinfection treatment and pain management. Tecovirimat was administered to 24% of the individuals, and 4% received post-exposure vaccinations. Finally, there were no fatalities reported.

**Figure 2:**
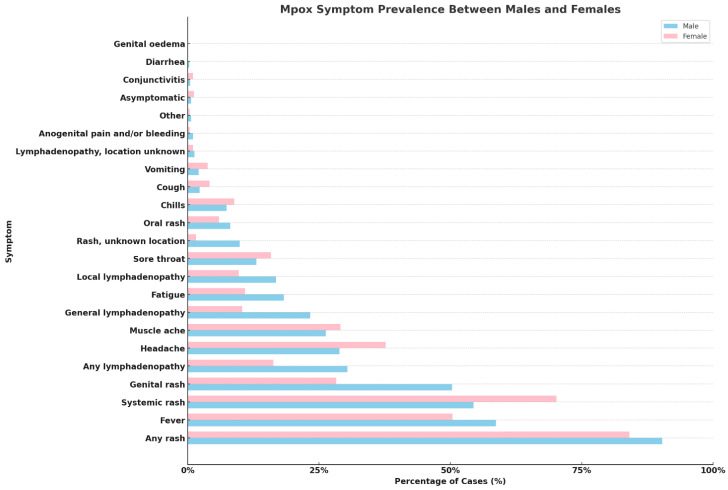
Comparative prevalence of Mpox symptoms in male and female patients.

Pooling all the data together, analyzing them, and sourcing from the WHO [18], some interesting sex- and gender-specific differences in Mpox symptom prevalence can be found (Figure 2). There are noticeable differences between genders in the rate of certain symptoms like “genital rash” and “any lymphadenopathy”, which show a higher prevalence in males compared to females, indicating possible variations in disease manifestation or reporting between genders. Some symptoms are, instead, common across genders, such as “any rash” and “fever”, even if slightly higher in males, suggesting that, while certain symptoms are universally common among Mpox patients, the extent to which they are experienced can still vary by gender. Some symptoms appear to be sex-/gender-specific with significant disparities, like “genital rash”, which is much more prevalent in males than in females, or “headache” and “muscle ache”, which, on the contrary, show a relatively higher prevalence in females. This could reflect differences in biological response, exposure, or even healthcare-seeking behaviors between males and females. Finally, symptoms, such as “conjunctivitis”, “diarrhea”, and “genital oedema”, are relatively rare in both genders, even though a few of them still present notable differences in prevalence between males and females.

### 3.3. Case Reports and Case Series

Sixteen studies [34,35,36,37,38,39,40,41,42,43,44,45,46,47,48,49,50] reporting eighteen cases were found and synthesized. The average age of patients ranged from 18 to 71 years. The transmission route was mostly sexual contact, with close contact and non-sexual routes being reported in the remaining cases. Antivirals (including treatments like tecovirimat and cidofovir) or symptomatic care (including symptomatic relief measures and topical treatments) was employed in a few instances, while, in the remaining cases, no treatment was necessary, or no detailed treatment information was provided. Overall, these clinical case reports and case series highlight the variability in transmission routes and treatment approaches for Mpox, as well as the broad age range of affected individuals (Table 3).

Ezzat et al. [34] described a 31-year-old female patient residing in Switzerland who presented to the gynecologic emergency department for painful vulvar lesions after an episode of an upper respiratory tract infection. Shortly after, the patient developed generalized and typical Mpox lesions on her whole body. She was initially misdiagnosed with a mycotic infection and later with genital herpes, before being correctly diagnosed with Mpox following the worsening of her symptoms and the appearance of additional lesions. The patient’s symptoms did not respond to antiviral or antifungal treatments, leading to further investigation and the eventual diagnosis of Mpox through PCR testing.

Vallée et al. [35] detailed the clinical case of sexually transmitted Mpox in a young French woman, characterized by distinctive genital lesions. In September 2022, this 18-year-old woman presented with a fever, myalgia, and a rash that began on her vulva seven days prior. The rash initially appeared in the gluteal area and subsequently spread to other parts of her body. A gynecological examination revealed ulcero-necrotic lesions around and within the vulva. The patient’s tests for other sexually transmitted infections, including HIV, syphilis, and hepatitis, were negative, as were those for her partner. However, a pharyngeal swab tested positive for the Mpox virus. The patient’s boyfriend had shown symptoms consistent with Mpox following a vacation, suggesting sexual transmission as the primary route of infection.

Bertoni et al. [36] highlighted the clinical case of a 27-year-old woman in a committed relationship, diagnosed with Mpox in August 2022 in Milan, Italy. She presented with a genital lesion for three days and had been experiencing prodromal symptoms like headaches, vulvodynia, and fatigue for ten days, with no history of international travel, HIV, or other sexually transmitted infections. A physical examination revealed right inguinal lymphadenopathy and a crusted lesion on her labia, prompting tests for Mpox through the lesion, oropharyngeal swabs, and blood samples. RT-PCR testing confirmed Mpox infection, with negative results for the oropharyngeal swab. The patient’s sexual partner, previously diagnosed with Mpox, likely transmitted the virus sexually.

Zayat et al. [37] reported the clinical case of a 22-year-old woman with no significant medical history, who visited the emergency department of a hospital in Brooklyn, New York, due to multiple painful lesions on her vulva and inside her vagina. She had engaged in vaginal intercourse with a male partner approximately 2.5 weeks earlier, during which she noticed several dark, bump-like lesions on his penis, resembling ingrown hairs. The partner’s sexual health status, including any sexually transmitted infections, was unknown. Two weeks post-intercourse, the patient began experiencing muscle pains, tiredness, and a fever. A couple of days following the fever’s onset, she observed a few mildly painful, flesh-colored bumps that turned white and multiplied the next day, leading her to seek medical help at another hospital. Despite initial treatment with lidocaine, bacitracin, and ibuprofen for pain management, her condition worsened, with the lesions enlarging and becoming more painful, especially when sitting, and spreading to her perianal area. By the fourth day, unable to bear the pain, she visited the emergency department. Her vital signs were stable, but the gynecological examination revealed various stages of lesions across her genital area, with no signs of lymphadenopathy or other skin lesions. Despite undergoing extensive testing for common sexually transmitted infections and a vulvar biopsy due to the unusual nature of the lesions, it was her partner’s subsequent positive Mpox virus diagnosis that led to her being tested specifically for the virus. She was then admitted and started a 14-day regimen of tecovirimat, following isolation guidelines. All her tests came back negative except for the Mpox virus, confirmed via PCR. After starting treatment with tecovirimat, she was discharged on the third day of hospitalization and reported significant improvement during a follow-up telehealth visit, with the lesions diminishing and the pain subsiding.

Cole et al. [38] highlighted a complex, multi-faceted case involving a 35-year-old White, apparently healthy woman from the UK, who developed encephalitis and longitudinally extensive transverse myelitis due to Mpox but showed a remarkable neurological recovery following treatment with antivirals (tecovirimat and cidofovir), analgesia, antibiotics for secondary infections, and ultimately, immunosuppressive therapy with steroids (methylprednisolone), and plasma exchange to manage the post-infectious autoimmune complications. Initially presenting with symptoms typical of a sexually transmitted infection after unprotected sex, the patient’s condition escalated to include severe genital lesions, systemic symptoms, and eventually significant neurological complications. The initial differential diagnosis included common causes of genital lesions like the herpes simplex virus and the varicella-zoster virus, but tests for these were negative. The diagnosis of Mpox was confirmed through PCR testing of the genital lesions, and the patient’s condition was complicated by severe pain, difficulty in urination, a systemic spread of the lesions, and lymphadenopathy. The situation became more critical with the development of neurological symptoms, leading to the suspicion and subsequent confirmation of Mpox encephalitis and later, longitudinally extensive transverse myelitis. With treatment, the patient’s condition, including the neurological deficits, showed improvement, highlighting the importance of multidisciplinary care in managing complex infectious disease presentations.

Mancha et al. [39] reported the unusual case of a 30-year-old female (Fitzpatrick phototype III), highlighting not only this disease’s potential to affect a broader population but also, and especially, a rather rare, previously undocumented transmission route, namely oro-mammary sex with a partner who had symptoms suggestive of tonsillitis (which was later confirmed to be Mpox). This emphasizes the viability of the virus in saliva and the potential for transmission through intimate, non-genital contact. From a clinical standpoint, the case started as an erythematous papule on the left nipple and evolved into a flat ulceration with a hemorrhagic crust surrounded by umbilicated pustules, along with systemic symptoms like a fever and lymphadenopathy, underscoring the diverse manifestations and transmission routes of Mpox in female individuals. The patient was treated with symptomatic care and topical fusidic acid and finally recovered from the infection.

Bruno et al. [40] presented a clinical case involving a 71-year-old Italian woman with no recent travel history but multiple sexual partners in the past year, who also had diabetes, obesity, hypertension, and bipolar disorder. She developed symptoms and a rash following sexual contact with a man who also had skin lesions but no recent travel history. Testing confirmed Mpox viral DNA in her lesions, but no antiviral treatment was necessary. This case is particularly noteworthy in that it reports a sexually transmitted infection in an older woman, with underlying comorbidities.

Sukhwani et al. [41] described the clinical case of a 36-year-old Caucasian woman, who sought medical attention at the emergency department due to painful, non-itchy vesicular lesions ranging from 3 to 8 mm across her pubic and vulvar regions, accompanied by painful pelvic lymphadenitis on the left side. Her partner, who was HIV-positive with an undetectable viral load, had lapsed in his medication regimen for a week during the past month. Initially believed to be a *Molluscum contagiosum*-virus infection, she was treated with topical podophyllotoxin cream. However, her condition deteriorated, leading to the development of a vulvar abscess and further vesicular lesions. Subsequent testing revealed a *Chlamydia trachomatis* infection, prompting the addition of Azithromycin and Acyclovir to her treatment regimen. Given the ongoing Mpox outbreak, a PCR test for Orthomyxovirus (Mpox) was conducted and returned positive. Despite having no travel history to affected regions, her close contact with an HIV-positive individual and past sexually transmitted diseases significantly raised her risk of infection. She was advised to isolate at home and showed complete recovery at a three-month follow-up.

Rai et al. [42] presented the clinical case of a woman aged 27 years, living with HIV, successfully maintaining suppressed HIV viremia through antiretroviral therapy (Biktarvy, which is a cocktail of bictegravir, emtricitabine, and tenofovir alafenamide), and with a history of hypothyroidism post-thyroidectomy for medullary thyroid cancer, who contracted Mpox. The authors thoroughly studied the effects of Mpox infection on the immune system. Despite clinically experiencing a facial rash and other mild systemic symptoms, significant changes were observed in her immune system, including alterations in B- and T-cell populations and various plasma biomarkers, indicating interactions between Mpox and HIV and a profound immunologic response to the infection. The patient’s immune profile was extensively analyzed, revealing notable changes such as increased levels of certain B cells, plasmablasts, and their immunoglobulin isotypes. Additionally, a significant rise in CD38+ HLA-DR+ CD8+ T cells was detected following the Mpox infection.

van Hennik and Petrignani [43] reported the case of a 57-year-old female, the partner of a bisexual man who tested positive for Mpox, presented at the Centre of Sexual Health at Den Haag, The Netherlands. During a physical examination, lesions characteristic of Mpox were observed at the vaginal opening. The patient reported experiencing symptoms for a period of eight days, beginning with itchiness and progressing to pain, which decreased after three days.

Napoli et al. [44] described a 28-year-old woman suffering from gastroesophageal reflux disease and untreated atopic dermatitis, who had just gotten a tattoo, and who presented with intense pain in her right ear and multiple vesiculopustular lesions. Within a week, she had developed around 80 lesions spread across her body. Lab tests confirmed an infection with the Mpox virus, and after starting treatment with oral tecovirimat, no new lesions appeared. The patient experienced severe pain, gastrointestinal distress, bacterial superinfection, acute kidney injury (AKI), and anemia as complications of Mpox. The use of tecovirimat, an antiviral approved for the treatment of Orthopoxvirus infections, was considered but posed challenges due to the patient’s AKI. This highlights the need for the careful consideration of treatment options in Mpox patients, especially those with comorbidities that may limit the use of certain medications. Moreover, this case emphasizes the importance of considering Mpox in differential diagnoses, even in the absence of known exposure or classic risk factors. It also highlights the need for heightened surveillance and preventive measures in settings where the virus may be present in the environment, such as tattoo parlors and piercing establishments. This case illustrates the ongoing need for research into Mpox, particularly regarding its transmission dynamics, clinical manifestations in diverse patient populations, and effective treatment options. The limited efficacy data for Mpox treatments and the challenges posed by comorbid conditions in affected individuals underline the importance of continued investigation and data collection.

Siedner et al. [45] reported the clinical case of a young woman, in her late twenties from the United States, who contracted Mpox without any known sexual or close physical contact with infected individuals in the two months prior to her diagnosis. The woman experienced a facial rash that began as itchy red spots on her face, which evolved into vesicles and then pustules. She had been prescribed doxycycline and valacyclovir initially but later started tecovirimat therapy when her condition did not improve. This case is particularly noteworthy due to the absence of traditional epidemiological risk factors. The woman lived alone and had not had any sexual or intimate contact for three months before the rash appeared. Her recent activities included business and leisure travel and visits to spas, where she received massages with her face resting on linens that could potentially have been contaminated. Public health investigations did not identify any Mpox cases among the spa staff or other clients, and both spas adhered to hygiene practices that included changing linens between clients and using disinfectants effectively active against enveloped viruses. The possibility of fomite transmission through contaminated linens or towels was considered, given the nature and location of her lesions, emphasizing the importance of strict hygiene practices in public settings such as spas. Moreover, the prolonged healing time of the lesions in this case also points to the necessity of further research on the duration of viral shedding from such ulcers.

Ogoina and James [46] presented a case involving a 24-year-old Nigerian female sex worker who tested positive for Mpox, which underscores the significance for public health in understanding the spread and management of Mpox within Africa and worldwide, especially in a socially vulnerable, highly stigmatized population, namely the community of sex workers. The patient began experiencing a fever and, four days after her last sexual encounter with a client in a brothel, developed vesiculopustular lesions on her groin and genital area.

Sampson et al. [47] presented a case of a 20-year-old pregnant woman at 31 weeks of gestation, with a history of sexually transmitted infections but no chronic conditions. She sought medical attention due to vaginal discharge, bleeding, painful urination, and decreased fetal movements for two weeks. At the genital exam, she presented with a new painful vaginal lesion (a 1 cm labial ulcer, affecting her left *labia majora*) and a subsequent herpes-like papular rash on her abdomen and leg at 31 weeks of gestation, along with tender lymph nodes in her left groin. She was admitted for a suspected urinary tract infection and fetal observation. Previously, she had been treated for gonorrhea, chlamydia, and pyelonephritis during her pregnancy. Initial screenings for HIV and syphilis were negative. Upon admission, she showed signs of tachycardia but no fever or high blood pressure, and fetal monitoring was normal. During the hospital stay, the vaginal lesion grew, accompanied by new, itchy, red lesions on her body. Six days after, she mentioned her partner had recently tested positive for Mpox and, on the seventh day, PCR tests confirmed her vaginal lesion was positive for Orthopoxvirus, while also indicating herpes simplex virus 1, suggesting viral shedding rather than the cause of the ulcer. After being diagnosed with Mpox infection and herpes co-infection, she was treated with tecovirimat and acyclovir. Her condition stabilized, with no new lesions, allowing for her discharge and for her to complete the tecovirimat treatment at home. Her lesions resolved 10 days after starting treatment. She had an uncomplicated induction of labor at 39 and 2/7 weeks of gestation and delivered a healthy neonate, who, despite reporting a temporary lesion on the scalp and having a positive immunoglobulin G test result for Orthopoxvirus, did not have skin lesions or positive molecular test results on the cord blood, fetal serum, maternal vaginal fluid, and the placenta’s surface that were suggestive of an infection. The baby remained healthy and developed normally at the three-month follow-up.

Renfro et al. [48] reported two cases of Mpox infection in pregnant, heterosexual cisgender women, focusing on their pregnancy and childbirth outcomes. Both women underwent labor induction and encountered complications from chorioamnionitis during childbirth. The first case is a 19-year-old female, in her first pregnancy, who experienced vaginal itching at 24 weeks of gestation. She tested negative for *Chlamydia trachomatis* and *Neisseria gonorrhoeae* but positive for Mpox from a vaginal swab. At 36 weeks, labor was induced due to intrahepatic cholestasis: during labor, she developed chorioamnionitis. Initial treatment for the presumed vaginitis included topical metronidazole, and labor was induced using a Cook balloon and an oxytocin infusion. Chorioamnionitis was treated aggressively with intravenous ampicillin and gentamicin. The second case was a 22-year-old female, also in her first pregnancy, who underwent routine sexually transmitted-infections screening at 36 weeks, testing negative for *C. trachomatis* and *N. gonorrhoeae* but positive for Mpox from a vaginal swab. At 38 weeks and 4 days, labor was induced due to oligohydramnios, which followed 48 h of fluid leakage. Similar to the first case, she developed chorioamnionitis during labor, which was induced with an oxytocin infusion. The treatment for chorioamnionitis mirrored that of the first case, with a regimen of intravenous ampicillin and gentamicin. Both cases illustrate the complexities of managing pregnant individuals with Mpox, especially when coupled with obstetric complications like intrahepatic cholestasis, oligohydramnios, and chorioamnionitis. The management strategies involved not only addressing the Mpox infection but also carefully navigating pregnancy complications to ensure the health and safety of both the mother and the fetus. Indeed, the use of antivirals like tecovirimat and vaccinia immune globulin in pregnant women may give rise to obstetric issues, such as the potential for a prolonged QT interval when corrected for heart rate, errors in measuring blood glucose levels, and an increased risk of venous thromboembolism caused by medical interventions.

Finally, Dung et al. [49] reported two women, who traveled from the United Arab Emirates to Vietnam, diagnosed with Mpox, hospitalized, and linked to a newer, emerging sub-lineage, A.2.1 (Clade IIb), differing from the B.1 lineage associated with the widespread outbreak. Patient 1, a 35-year-old woman, exhibited symptoms after sexual contact in Dubai, including a fever and a maculopapular rash. She tested positive for Mpox and the varicella-zoster virus. Patient 2, a 38-year-old woman and friend of Patient 1, also showed symptoms following a sexual encounter in Dubai and tested positive for Mpox upon her return to Vietnam. Both patients were afebrile upon admission and had stable conditions throughout their hospitalization. They were isolated according to local health regulations. Patient 1 was treated with oral acyclovir due to the varicella-zoster virus co-infection. No specific treatments were mentioned for Patient 2. This interesting case series suggests women may also play a role in transmitting Mpox, underscoring the importance of advanced genomic monitoring to understand the virus’s evolution. More in detail, the phylogenetic analysis of the Mpox viral strains from the patients detected a novel nonsynonymous substitution from threonine to isoleucine in amino 717 (T717I mutation) in the polymerase protein, which was identified in Patient 1’s virus sequence, indicating potential genetic diversity within the strains. This case series has major epidemiological and public health implications, highlighting the role of women in Mpox transmission networks and the need for enhanced genomic surveillance to understand and monitor the epidemiology and evolution of the Mpox virus.

### 3.4. Case Series Review

Schwartz and Pittman [50] reviewed 58 cases of pregnant women positive for Mpox infection that were reported during the 2022–2023 outbreak. These cases include the 23 cases reported by Oakley et al. [26]. There have been no documented cases of negative outcomes related to pregnancies or childbirths, such as stillbirths. The lack of complications during pregnancies or at birth linked to Clade IIb is consistent with the overall mortality rate of less than 0.1% among non-pregnant women, as infections from this clade tend to be milder compared to those caused by the Clade I or IIa variants of Mpox. Therefore, the two researchers formulated a hypothesis according to which Mpox viral clade differences may be associated with varying obstetrical and fetal outcomes.

## 4. Discussion

During the 2022–2023 multi-country outbreak, Mpox primarily affected cisgender men, especially those having sex with men, with fewer cases in women [51]. This review aimed to address the knowledge gap regarding gender-specific characteristics of Mpox by reviewing the scientific literature on infections in women, including diverse cases and geographical locations. Despite the lesser involvement of women, we could note specific trends and outcomes in the female cases studied. Women diagnosed with Mpox presented differences in epidemiological, behavioral, and clinical characteristics compared to men.

For instance, the patient described by Napoli et al. [44] exhibited rather unusual clinical features, including *erythema multiforme* and lesions within a tattoo, which are not commonly associated with Mpox, underscoring the variability in Mpox presentations, especially in individuals with underlying skin conditions like atopic dermatitis, which may alter the typical progression and appearance of this disease. Also, this case raises questions about the transmission dynamics of Mpox, as the patient had no known direct contact with Mpox cases, suggesting the possibility of an indirect transmission or acquisition from environmental sources. This appears to be supported by a few reports [52,53,54,55,56], which found Mpox transmissions in tattoo parlors and piercing establishments, indicating that the virus can persist in the environment and infect individuals through less direct routes.

Health services should provide a comprehensive assessment that accounts for gender diversity and should promote international collaborations in monitoring and managing infectious diseases. Our findings underscore, indeed, the necessity of inclusive, tailored surveillance and research to understand the dynamics of Mpox across different population groups, including women and those who are pregnant.

Specifically concerning pregnancy, this topic has been particularly underexplored in the currently available body of scholarly literature. Only a few cases have been reported, including those from previous outbreaks. For instance, Mbala et al. [57] documented the pregnancy outcomes of four expectant mothers included in a study conducted at the General Hospital of Kole (Sankuru Province), DRC. This study observed 222 individuals presenting symptoms between 2007 and 2011. Among these four pregnant participants, one delivered a healthy baby, two experienced first-trimester miscarriages, and one reported a fetal demise. The deceased fetus, which was macerated, exhibited widespread maculopapular skin lesions covering the head, body, and limbs, extending to the palms and soles. Schwartz et al. [58] described the autopsy findings of a stillborn fetus at 21 weeks, diagnosed with congenital Mpox syndrome in the DRC in 2008. The infection was transmitted from the mother to the fetus via the placenta, and Mpox virus presence in the mother, fetus, and placenta was confirmed through an Mpox virus-specific quantitative PCR, even if the virus subtype could not be identified.

A histological analysis of placentas from pregnancies affected by congenital cowpox and smallpox, viruses closely related to Mpox, revealed the presence of intracytoplasmic inclusions called Guarnieri bodies in the decidua, along with documented signs of viral cytopathic effects, such as granulomas, inflammation, and necrotic placental villi. Taken altogether, these observations suggest that Orthopoxviruses may be capable of crossing the placental barrier, though the specific processes involved are still not fully understood [59,60]. Dashraath et al. [60] have proposed four possible pathways of in utero transmissions, namely (i) ascension from the genital tract (vagina, cervix, and fluids) to the placenta (chorionic membranes, including epithelial and mesenchymal cells and trophoblasts); (ii) a spread via the bloodstream, reaching the placenta through the spiral arteries in the uterus, then infecting the decidua, extra-villous trophoblast, or the placental villi, and, finally, entering the fetal circulation; (iii) a direct infection of placental cells (a direct infection of the syncytiotrophoblast by transcytosis or a fusion with the trophoblast membrane); and (iv) a viral invasion facilitated by inflammation (with innate and adaptive maternal immune responses in the decidua releasing cytokines which disrupt the cortical actin network overlying the syncytiotrophoblast and/or Mpox-induced type 1 interferon responses via the release of IFN-α or IFN-β antiviral mediators). All this suggests complex risks and mechanisms of Mpox transmission during pregnancies [60].

Given the paucity of information, experts [61,62,63,64,65] recommend that pregnant, postpartum, and lactating women should wear masks, particularly when in the vicinity of potentially infected individuals, and should avoid contact with anyone displaying symptoms like a fever or skin lesions on mucous membranes. Moreover, they should practice safe sex using condoms during oral, vaginal, and anal intercourse due to the high risk of transmission through intimate contact. They should be vigilant for any genital lesions in their sexual partner and seek medical consultation immediately upon noticing any concerning symptoms to facilitate a timely clinical and laboratory diagnosis. Healthcare professionals should advise pregnant women with a mild illness to isolate at home with regular monitoring by their healthcare team and ensure that cases with severe symptoms receive in-hospital care. The absence of a specific antiviral treatment protocol for the pregnancy–puerperal cycle should be noted. Close attention should be paid to monitoring fetal well-being in patients with moderate to severe illnesses due to increased risks of fetal complications. Healthcare professionals should make obstetric decisions regarding deliveries on a case-by-case basis, with cesarean sections not routinely recommended, and should advise delaying breastfeeding during isolation, providing support for re-lactation afterwards.

Even less data are available on Mpox among transgender women and non-binary individuals assigned to female sex at birth. This lack of data presents challenges in understanding the full scope and impact of this disease within these groups, who may have unique health needs and risk factors compared to the broader population. Transgender women and non-binary individuals often face barriers in accessing healthcare, including discrimination, a lack of provider knowledge on gender-diverse health needs, and economic constraints. These challenges can lead to the underreporting of health issues and decreased participation in health studies, further contributing to the scarcity of data. Additionally, the social and behavioral factors that affect the risk of Mpox transmission in these communities might differ from those in the general population. For instance, the networks and dynamics of sexual partnerships, the prevalence of other sexually transmitted infections which may facilitate Mpox transmission, and the use of gender-affirming medical interventions that might impact immune function are all areas that require targeted research. Given these complexities, there is a critical need for more inclusive and comprehensive research efforts. Studies should be designed to explicitly include and address the health concerns of transgender women and non-binary individuals, taking into account the diverse experiences and challenges they face. Improving data collection and research methodologies to be more inclusive of gender diversity will not only help in understanding the epidemiology of Mpox in these groups but will also inform more effective public health strategies and interventions tailored to their needs.

The present review emphasizes the importance of considering gender and sexual orientation in public health responses to effectively address the spread and impact of this disease. For this purpose, inclusively collecting data on sex, gender identity and expression, and sexual orientation on a routine basis would be essential. Further research is encouraged to explore the nuances of transmission, behavior, and disease progression among diverse groups, enhancing the overall response to Mpox and similar public health challenges (Table 4).

## 5. Future Directions

In-depth studies on Mpox transmission dynamics in women, particularly focusing on non-sexual routes and environmental factors, are warranted to better understand the full spectrum of transmission risks and provide guidance over the pharmacological aspects, since data on antivirals in women and specific populations, such as pregnant or breastfeeding women, are lacking. An exploration of the unique clinical manifestations of Mpox in women, especially in those with underlying health conditions, is necessary to tailor clinical management and public health interventions. Enhanced surveillance and research on Mpox in pregnant women are highly needed, in order to systematically document outcomes and provide evidence-based guidelines for management during pregnancies. An investigation into Mpox’s impact on transgender women and non-binary individuals assigned female at birth should address the lack of data and the specific health needs and risk factors of these populations. Finally, genomic monitoring and analysis should be implemented to track the evolution of the Mpox virus, particularly in light of cases linked to new sub-lineages, which could have implications for vaccine and treatment efficacy, especially among cisgender and transgender women and non-binary individuals assigned to the female sex at birth.

## 6. Conclusions

This comprehensive review of Mpox’s impact on women, across various global contexts, underlines this disease’s distinct epidemiological and clinical manifestations in female populations, including pregnant women. Despite the predominance of cases among cisgender men, particularly those having sex with men, the findings from cohort studies, case series, case reports, and a literature review highlight the necessity for gender-inclusive surveillance and research. This approach is crucial to understand and address the unique risks and outcomes associated with Mpox in women, advocating for tailored public health strategies and interventions that consider gender diversity and the specific needs of women and pregnant individuals in managing infectious diseases.

## Figures and Tables

**Table 1:** The systematic review criteria for investigating Mpox in women: a PECOS-based framework with an overview of the search methodology.

Search Criteria	Brief Description
Population	Women affected by Mpox, including cisgender and transgender women and non-binary individuals assigned female at birth
Exposure	Infection with Mpox virus
Comparator	Men (either cisgender or transgender and non-binary individuals assigned male at birth)
Outcome	Clinical manifestations of Mpox, epidemiological features, transmission routes, and treatment responses
Study Design	Cohort studies, case reports, case series, cross-sectional studies, and online surveys focusing on Mpox in the specified population
Keywords	(Monkeypox OR Mpox) AND (women OR woman OR female OR females OR male-to-female OR transwoman OR transwomen OR pregnant OR pregnancy OR lactation OR breastfeeding OR postpartum)
Databases Searched	MEDLINE/PubMed, Scopus, Web of Science, and EMBASE
Hand-searched target journals	AJOG Glob Rep: Emerg Infect Dis, Enferm Infecc Microbiol Clin, Eur J Obstet Gynecol Reprod Biol, Euro Surveill, IDCases, IJID Reg, J Eur Acad Dermatol Venereol, J Infect Dis, Lancet, J Med Virol, Lancet Infect Dis, MMWR Morb Mortal Wkly Rep, Medicina (B Aires), Ned Tijdschr Geneeskd, Obstet Gynecol, Open Forum Infect Dis, Travel Med Infect Dis, and Viruses
Gray literature	WHO, PHAC, CDC, ECDC, and UKHSA

**Table 2:** A comparative analysis of Mpox’s clinical and epidemiological patterns in women: a global perspective.

Study	Study Location	Study Type	Participant Details	Main Findings	Specific Observations
Oakley et al. [28]	USA	Cohort study.	769 cisgender women, including 23 pregnant individuals (21 cases of Mpox during pregnancy and 2 within 3 weeks of pregnancy).	Predominant impact on specific ethnic groups; sexual or intimate contact as primary transmission route.	Cases among pregnant women; some required hospitalization.
Sánchez Doncell et al. [29]	Argentina	Retrospective analysis.	3 women, including 2 cisgender women and 1 transgender woman.	Low incidence among women; focus was on sexual health impacts.	No complications reported; symptoms included headaches, myalgias, and a fever.
Coutinho et al. [30]	Brazil	Surveillance data.	108 women (cisgender and transgender) and 10 non-binary persons.	Older women, more non-sexual contact, fewer genital lesions, and lower HIV prevalence compared to men.	Hospitalizations but no deaths among women.
Grothe et al. [31]	Europe	Online survey.	Women across Spain and Belgium, among others.	Higher likelihood of infection in Spain and Portugal.	Disparities in diagnosis delays and clinical manifestations between genders.
Vallejo-Plaza et al. [32]	Spain	Surveillance data analysis.	Both men and women with Mpox cases reported in Spain.	Women constituted 2.1% of the total Mpox cases, showing a younger median age compared to men. The primary route of transmission was close contact during sexual relations for both genders, but women also had significant other transmission routes.	Women experienced longer diagnosis delays. Women showed different symptom patterns and risk profiles.
Thornhill et al. [33]	Global (15 countries)	Data collection.	136 cisgender and transgender women, non-binary individuals.	High HIV prevalence among trans women; many contracted the virus through sexual contact.	Misdiagnosis in a significant portion; majority presented with anogenital rash.
Ezzat et al. [34]; Vallée et al. [35]; Bertoni et al. [36] Zayat et al. [37]; Cole et al. [38]; Mancha et al. [39]; Bruno et al. [40]; Sukhwani et al. [41]; Rai et al. [42]; van Hennik and Petrignani [43]; Napoli et al. [44]; Siedner et al. [45]; Ogoina and James [46]; Sampson et al. [47]; Renfro et al. [48]; and Dung et al. [49]	Globally (various case reports)	Case reports.	Individual women cases.	Unusual transmission routes; severe complications in some cases.	Diversity in clinical manifestations, and transmission routes highlighted.
Schwartz and Pittman [50]	Globally (various case reports)	Review of 58 cases.	Cases of pregnant women positive for Mpox infection during the 2022–2023 outbreak.	No documented cases of negative outcomes. Absence of complications linked to Mpox Clade IIb.	Mpox clades could influence the severity of the infection and its impact on pregnancies and fetal health.

**Table 3:** Comprehensive overview of diverse Mpox cases among women: transmission, clinical presentation, and treatment outcomes.

Study	Patient Profile	Transmission Route	Clinical Presentation	Treatment and Outcome
Ezzat et al. [34]	Thirty-one-year-old female in Switzerland.	Sexual transmission.	Painful vulvar lesions and generalized Mpox lesions.	Initial misdiagnosis; confirmed Mpox through PCR; treatment details not specified.
Vallée et al. [35]	Eighteen-year-old French woman.	Sexual transmission from her boyfriend diagnosed with Mpox.	Fever, myalgia, and rash on vulva, spreading to other body parts, and ulcero-necrotic lesions around and within the vulva.	Pharyngeal swab tested positive for Mpox virus.
Bertoni et al. [36]	Twenty-seven-year-old woman in Milan, Italy.	Sexual transmission from her sexual partner diagnosed with Mpox.	Genital lesions, headaches, vulvodynia, fatigue, right inguinal lymphadenopathy, and crusted lesions on labia.	Mpox confirmed by RT-PCR testing.
Zayat et al. [37]	Twenty-two-year-old American woman.	Sexual transmission from a male partner with lesions on his penis.	Multiple painful lesions on vulva and inside vagina, muscle pains, tiredness, fevers.	Initially treated for pain management. After partner’s Mpox diagnosis, confirmed positive for Mpox via PCR. Started on a 14-day regimen of tecovirimat and discharged on the third day of hospitalization. Significant improvement reported during follow-up, with lesions diminishing and pain subsiding.
Cole et al. [38]	Thirty-five-year-old White, apparently healthy woman from the UK.	Unprotected sex.	Severe genital lesions, systemic symptoms, encephalitis, and longitudinally extensive transverse myelitis.	Antivirals (tecovirimat and cidofovir), analgesia, antibiotics, steroids, and plasma exchange; remarkable neurological recovery.
Mancha et al. [39]	Thirty-year-old female, Fitzpatrick phototype III.	Oro-mammary sex.	Erythematous papule on left nipple, evolving into flat ulceration with hemorrhagic crust and umbilicated pustules; fevers and lymphadenopathy.	Symptomatic care and topical fusidic acid; recovered.
Bruno et al. [40]	Seventy-one-year-old Italian woman with diabetes, obesity, hypertension, bipolar disorder, and multiple sexual partners.	Sexual contact with a man who had skin lesions.	Symptoms and rashes developed after sexual contact.	Mpox viral DNA confirmed in lesions. No antiviral treatment was necessary.
Sukhwani et al. [41]	Thirty-six-year-old Caucasian woman, partner is HIV-positive.	Close contact with an HIV-positive individual.	Painful, non-itchy vesicular lesions on pubic and vulvar regions and painful pelvic lymphadenitis.	Initially treated for *Molluscum contagiosum* with topical podophyllotoxin. Condition deteriorated. Added Azithromycin and Acyclovir after *Chlamydia trachomatis* infection was detected. Mpox confirmed by PCR. Advised home isolation and showed complete recovery at three-month follow-up.
Rai et al. [42]	Twenty-seven-year-old woman living with HIV, history of hypothyroidism post-thyroidectomy for medullary thyroid cancer.	Sexual contact.	Facial rash and mild systemic symptoms observed.	This study focused on the immunologic response to Mpox infection, revealing notable changes in the immune profile, including alterations in B- and T-cell populations and various plasma biomarkers.
van Hennik and Petrignani [43]	Fifty-seven-year-old female, partner of a bisexual man.	Close contact.	Lesions at vaginal opening.	Symptoms for a period of eight days, beginning with itchiness and progressing to pain, decreasing after three days.
Napoli et al. [44]	Twenty-eight-year-old woman with gastroesophageal reflux, untreated atopic dermatitis.	Recent tattoo.	Intense ear pain and multiple vesiculopustular lesions.	Oral tecovirimat; complications included pain, GI distress, bacterial superinfection, AKI, and anemia.
Siedner et al. [45]	Young woman in her late twenties from the United States, living alone, no known close physical contact with infected individuals.	Possible fomite transmission through contaminated linens at spas.	Facial rash, evolving from itchy red spots to vesicles and pustules.	Initially prescribed doxycycline and valacyclovir and later started on tecovirimat therapy.
Ogoina and James [46]	Twenty-four-year-old Nigerian female sex worker.	Sexual contact in a female sex worker.	Fever and vesiculopustular lesions on groin and genital area.	Symptoms developed four days after the last sexual encounter
Sampson et al. [47]	Twenty-year-old pregnant woman at 31 weeks of gestation.	Sexual contact.	Vaginal discharge, bleeding, painful urination, labial ulcer, and herpes-like rash.	Tecovirimat and acyclovir; stable condition, discharged, and lesions resolved.
Renfro et al. [48]	Two pregnant, heterosexual cisgender women.	During pregnancy.	Vaginal itching, chorioamnionitis during childbirth.	Induced labor, antibiotics for chorioamnionitis.
Dung et al. [49]	Two women, 35 and 38 years old, who traveled from UAE to Vietnam.	Sexual contact.	Fever and maculopapular rash.	Isolation and oral acyclovir for co-infection in Patient 1

**Table 4:** Strategic directions for advancing Mpox research and public health interventions in women and gender-diverse populations.

Research Area	Recommendations	Suggestions
Transmission Dynamics	Conduct studies focusing on non-sexual transmission routes and environmental factors.	Collaborate with community organizations to reach diverse populations.
Clinical Manifestations.	Investigate unique clinical manifestations in women, especially those with underlying health conditions.	Develop specialized training for healthcare providers on recognizing and managing Mpox in women.
Mpox in Pregnancy	Systematically document Mpox outcomes in pregnant women to inform management guidelines.	Engage obstetric and gynecological associations to develop and disseminate guidelines.
Impact on Transgender Women and Non-Binary Individuals	Address the lack of data for transgender women and non-binary individuals, focusing on their specific health needs.	Include transgender and non-binary individuals in research and public health campaigns.
Genomic Monitoring	Implement genomic analysis to track virus evolution and its implications for treatment and vaccine efficacy.	Coordinate with international health organizations for data sharing and joint research initiatives.

## Data Availability

No new data were generated.

## References

[1] Bunge E.M., Hoet B., Chen L., Lienert F., Weidenthaler H., Baer L.R., Steffen R. (2022). The changing epidemiology of human monkeypox-A potential threat? A systematic review. PLoS Negl. Trop. Dis..

[2] Muyembe-Tamfum J.J., Mulembakani P., Lekie R.B., Szczeniowski M., Ježek Z., Doshi R., Hoff N., Rimoin A.W. (2011). Smallpox and its eradication in the Democratic Republic of Congo: Lessons learned. Vaccine.

[3] Rimoin A.W., Mulembakani P.M., Johnston S.C., Lloyd Smith J.O., Kisalu N.K., Kinkela T.L., Blumberg S., Thomassen H.A., Pike B.L., Fair J.N. (2010). Major increase in human monkeypox incidence 30 years after smallpox vaccination campaigns cease in the Democratic Republic of Congo. Proc. Natl. Acad. Sci. USA.

[4] Thornhill J.P., Gandhi M., Orkin C. (2023). Mpox: The Reemergence of an Old Disease and Inequities. Annu. Rev. Med..

[5] Ahmed S.K., Mohamed M.G., Dabou E.A., Abuijlan I., Chandran D., El-Shall N.A., Chopra H., Dhama K. (2023). Monkeypox (Mpox) in immunosuppressed patients. F1000Research.

[6] Ogoina D., Damon I., Nakoune E. (2023). Clinical review of human Mpox. Clin. Microbiol. Infect..

[7] Islam M.M., Dutta P., Rashid R., Jaffery S.S., Islam A., Farag E., Zughaier S.M., Bansal D., Hassan M.M. (2023). Pathogenicity and virulence of monkeypox at the human-animal-ecology interface. Virulence.

[8] Chauhan R.P., Fogel R., Limson J. (2023). Overview of Diagnostic Methods, Disease Prevalence and Transmission of Mpox (Formerly Monkeypox) in Humans and Animal Reservoirs. Microorganisms.

[9] Hatmal M.M., Al-Hatamleh M.A.I., Olaimat A.N., Ahmad S., Hasan H., Ahmad Suhaimi N.A., Albakri K.A., Abedalbaset Alzyoud A., Kadir R., Mohamud R. (2022). Comprehensive literature review of monkeypox. Emerg. Microbes Infect..

[10] Zebardast A., Latifi T., Shafiei-Jandaghi N.Z., Gholami Barzoki M., Shatizadeh Malekshahi S. (2023). Plausible reasons for the resurgence of Mpox (formerly Monkeypox): An overview. Trop. Dis. Travel. Med. Vaccines.

[11] World Health Organization (WHO) WHO Director-General Declares the Ongoing Monkeypox Outbreak a Public Health Emergency of International Concern. 23 July 2022. https://www.who.int/europe/news/item/23-07-2022-who-director-general-declares-the-ongoing-monkeypox-outbreak-a-public-health-event-of-international-concern.

[12] Karagoz A., Tombuloglu H., Alsaeed M., Tombuloglu G., AlRubaish A.A., Mahmoud A., Smajlović S., Ćordić S., Rabaan A.A., Alsuhaimi E. (2023). Monkeypox (Mpox) virus: Classification, origin, transmission, genome organization, antiviral drugs, and molecular diagnosis. J. Infect. Public Health.

[13] Borges V., Duque M.P., Martins J.V., Vasconcelos P., Ferreira R., Sobral D., Pelerito A., de Carvalho I.L., Núncio M.S., Borrego M.J. (2023). Viral genetic clustering and transmission dynamics of the 2022 Mpox outbreak in Portugal. Nat. Med..

[14] Bragazzi N.L., Kong J.D., Wu J. (2023). Integrated epidemiological, clinical, and molecular evidence points to an earlier origin of the current monkeypox outbreak and a complex route of exposure. J. Med. Virol..

[15] Limonta S., Lapadula G., Mezzadri L., Corsico L., Rovida F., Ranzani A., Baldanti F., Bonfanti P. (2024). Mpox Virus in the Pharynx of Men Having Sex with Men: A Case Series. Pathogens.

[16] Thornhill J.P., Barkati S., Walmsley S., Rockstroh J., Antinori A., Harrison L.B., Palich R., Nori A., Reeves I., Habibi M.S. (2022). Monkeypox Virus Infection in Humans across 16 Countries—April–June 2022. N. Engl. J. Med..

[17] Bragazzi N.L., Kong J.D., Mahroum N., Tsigalou C., Khamisy-Farah R., Converti M., Wu J. (2023). Epidemiological trends and clinical features of the ongoing monkeypox epidemic: A preliminary pooled data analysis and literature review. J. Med. Virol..

[18] World Health Organization (WHO) (2023). 2022 Mpox (Monkeypox) Outbreak: Global Trends. https://worldhealthorg.shinyapps.io/mpx_global.

[19] Lawry L.L., Lugo-Robles R., McIver V. (2023). Overlooked sex and gender aspects of emerging infectious disease outbreaks: Lessons learned from COVID-19 to move towards health equity in pandemic response. Front. Glob. Womens Health.

[20] Alvarez-Gómez A.M., Vélez-Cuervo S.M., Cardona-Maya W.D. (2023). Monkey pox: Importance in female sexual health. Rev. Colomb. Obstet. Ginecol..

[21] Ghebreyesus T.A., Allotey P., Narasimhan M. (2024). Advancing the “sexual” in sexual and reproductive health and rights: A global health, gender equality and human rights imperative. Bull. World Health Organ..

[22] Hoenig L.J. (2022). The Monkeypox Outbreak 2022: Women and Children Patients. Skinmed.

[23] Khalil A., Samara A., Ladhani S., O’Brien P. (2022). Monkeypox and pregnancy: Time for global surveillance and prevention strategies. Lancet.

[24] Pomar L., Favre G., Baud D. (2022). Monkeypox infection during pregnancy: European registry to quantify maternal and fetal risks. Ultrasound Obstet. Gynecol..

[25] Rodriguez-Morales A.J., Amer F.A. (2022). Monkeypox virus infection in women and non-binary people: Uncommon or neglected?. Lancet.

[26] Moher D., Shamseer L., Clarke M., Ghersi D., Liberati A., Petticrew M., Shekelle P., Stewart L.A., PRISMA-P Group (2015). Preferred reporting items for systematic review and meta-analysis protocols (PRISMA-P) 2015 statement. Syst. Rev..

[27] Page M.J., McKenzie J.E., Bossuyt P.M., Boutron I., Hoffmann T.C., Mulrow C.D., Shamseer L., Tetzlaff J.M., Akl E.A., Brennan S.E. (2021). *The PRISMA* 2020 statement: An updated guideline for reporting systematic reviews. BMJ.

[28] Oakley L.P., Hufstetler K., O’Shea J., Sharpe J.D., McArdle C., Neelam V., Roth N.M., Olsen E.O., Wolf M., Pao L.Z. (2023). Mpox Cases Among Cisgender Women and Pregnant Persons—United States, May 11–November 7, 2022. MMWR Morb. Mortal Wkly. Rep..

[29] Sánchez Doncell J., Lemos M., Francos J.J.L., González Montaner P. (2024). Viruela símica: Características en población femenina, Buenos Aires, Argentina [Monkeypox: Characteristics in female population, Buenos Aires, Argentina]. Medicina.

[30] Coutinho C., Secco Torres Silva M., Torres T.S., Peixoto E., Avelar Magalhães M., Wagner Cardoso S., Nazário G., Mendonça M., Menezes M., Almeida P.M. (2023). Characteristics of women diagnosed with Mpox infection compared to men: A case series from Brazil. Travel. Med. Infect. Dis..

[31] Grothe J.H., Cornely O.A., Salmanton-García J., VACCELERATE Consortium (2023). Monkeypox in children and adult women in Europe: Results from a flash VACCELERATE pilot survey. Enferm. Infecc. Microbiol. Clin. (Engl. Ed.).

[32] Vallejo-Plaza A., Rodríguez-Cabrera F., Hernando Sebastián V., Guzmán Herrador B.R., Santágueda Balader P., García San Miguel Rodríguez-Alarcón L., Díaz Franco A., Garzón Sánchez A., Sierra Moros M.J., Spanish Monkeypox Response Network (2022). Mpox (formerly monkeypox) in women: Epidemiological features and clinical characteristics of Mpox cases in Spain, April to November 2022. Euro Surveill..

[33] Thornhill J.P., Palich R., Ghosn J., Walmsley S., Moschese D., Cortes C.P., Galliez R.M., Garlin A.B., Nozza S., Mitja O. (2022). Human monkeypox virus infection in women and non-binary individuals during the 2022 outbreaks: A global case series. Lancet.

[34] Ezzat D., Barcellini B., Meier J., Duc-Ha E., Mathis J. (2023). Ulcerating vulvar lesions revealing a rare female case of monkeypox in Switzerland. AJOG Glob. Rep..

[35] Vallée A., Chatelain A., Carbonnel M., Racowsky C., Fourn E., Zucman D., Ayoubi J.M. (2023). Monkeypox Virus Infection in 18-Year-Old Woman after Sexual Intercourse, France, September 2022. Emerg. Infect. Dis..

[36] Bertoni C., Raccagni A.R., Candela C., Bruzzesi E., Mileto D., Canetti D., Rizzo A., Morsica G., Castagna A., Nozza S. (2023). Beyond stigma: Monkeypox infection in a 27-year-old woman. J. Med. Virol..

[37] Zayat N., Huang S., Wafai J., Philadelphia M. (2023). Monkeypox Virus Infection in 22-Year-Old Woman after Sexual Intercourse, New York, USA. Emerg. Infect. Dis..

[38] Cole J., Choudry S., Kular S., Payne T., Akili S., Callaby H., Gordon N.C., Ankcorn M., Martin A., Hobson E. (2023). Monkeypox encephalitis with transverse myelitis in a female patient. Lancet Infect. Dis..

[39] Mancha D., Brazão C., Filipe P. (2023). Oro-mammary inoculation pathway of monkeypox in a female patient. J. Eur. Acad. Dermatol. Venereol..

[40] Bruno G., Fabrizio C., Rodano L., Buccoliero G.B. (2023). Monkeypox in a 71-year-old woman. J. Med. Virol..

[41] Sukhwani M., Labrado P., Isla R., Segura A., José Escribano J. (2023). A case report of Monkeypox in a 36-year old woman in Madrid. Eur. J. Obstet. Gynecol. Reprod. Biol..

[42] Rai M.A., Shi V., Kennedy B.D., Justement J.S., Manning M.R., Praiss L., Kang E.J., Gittens K., Kardava L., Blazkova J. (2023). Impact of Monkeypox Virus Infection on Immune Parameters in a Woman with Human Immunodeficiency Virus Receiving Clinically Effective Antiretroviral Therapy. J. Infect. Dis..

[43] van Hennik M.M., Petrignani M.W.F. (2022). Klinische presentatie van monkeypox bij de vrouw [Clinical presentation of monkeypox in women]. Ned. Tijdschr. Geneeskd..

[44] Napoli E., Frizzell M., Gravell C., Vallejo S., Theodore S., Chen K., Siddiqui H., Dunn J., Marrufo D., Cadena J. (2023). Eczema Monkeypoxicum in a Female Patient with Atopic Dermatitis. Open Forum Infect. Dis..

[45] Siedner M.J., Trinidad J., Berto C.G., Brown C.M., Madoff L.C., Lee E.H., Iqbal M., Samson O., Albin J., Turbett S.E. (2023). Mpox in Young Woman with No Epidemiologic Risk Factors, Massachusetts, USA. Emerg. Infect. Dis..

[46] Ogoina D., James I.H. (2022). Mpox in a female sex worker in Nigeria: A case report. IJID Reg..

[47] Sampson M.M., Magee G., Schrader E.A., Dantuluri K.L., Bukhari A., Passaretti C., Temming L., Leonard M., Philips J.B., Weinrib D. (2023). Mpox (Monkeypox) Infection During Pregnancy. Obstet. Gynecol..

[48] Renfro Z.T., Contag C.A., Lu J., Solis D., Huang C., Sahoo M.K., Yamamoto F., Mah J., Jones M.S., Lin J. (2023). Two cases of MPXV infection during pregnancy in heterosexual cisgender women without classic cutaneous lesions, Northern California, 2022. IDCases.

[49] Dung N.T., Hung L.M., Hoa H.T.T., Nga L.H., Hong N.T.T., Thuong T.C., Ngoc N.M., Ny N.T.H., Quy V.T., Thoa V.T.K. (2023). Monkeypox Virus Infection in 2 Female Travelers Returning to Vietnam from Dubai, United Arab Emirates, 2022. Emerg. Infect. Dis..

[50] Schwartz D.A., Pittman P.R. (2023). Mpox (Monkeypox) in Pregnancy: Viral Clade Differences and Their Associations with Varying Obstetrical and Fetal Outcomes. Viruses.

[51] Bragazzi N.L., Khamisy-Farah R., Tsigalou C., Mahroum N., Converti M. (2023). Attaching a stigma to the LGBTQI+ community should be avoided during the monkeypox epidemic. J. Med. Virol..

[52] Tascini C., Geminiani M., Sbrana F., Pagotto A., Martini L. (2022). Possible tattoo-transmitted monkeypox viral infection. Intern. Emerg. Med..

[53] Del Río García V., Palacios J.G., Morcillo A.M., Duran-Pla E., Rodríguez B.S., Lorusso N. (2022). Monkeypox outbreak in a piercing and tattoo establishment in Spain. Lancet Infect. Dis..

[54] Viedma-Martinez M., Dominguez-Tosso F.R., Jimenez-Gallo D., Garcia-Palacios J., Riera-Tur L., Montiel-Quezel N., Linares-Barrios M. (2023). MPXV Transmission at a Tattoo Parlor. N. Engl. J. Med..

[55] Beaumont A.L., Raphaël E., Bertin C., Lariven S., Peiffer-Smadja N. (2023). Mpox lesions on a tattoo. Lancet Infect. Dis..

[56] Tascini C., Sbrana F., Giuliano S., Geminiani M., Pagotto A. (2023). Monkeypox virus transmission in tattoo parlor. New Microbiol..

[57] Mbala P.K., Huggins J.W., Riu-Rovira T., Ahuka S.M., Mulembakani P., Rimoin A.W., Martin J.W., Muyembe J.T. (2017). Maternal and Fetal Outcomes Among Pregnant Women with Human Monkeypox Infection in the Democratic Republic of Congo. J. Infect. Dis..

[58] Schwartz D.A., Mbala-Kingebeni P., Patterson K., Huggins J.W., Pittman P.R. (2023). Congenital Mpox Syndrome (Clade I) in Stillborn Fetus after Placental Infection and Intrauterine Transmission, Democratic Republic of the Congo, 2008. Emerg. Infect. Dis..

[59] Schwartz D.A., Ha S., Dashraath P., Baud D., Pittman P.R., Adams Waldorf K.M. (2023). Mpox Virus in Pregnancy, the Placenta, and Newborn. Arch. Pathol. Lab. Med..

[60] Dashraath P., Alves M.P., Schwartz D.A., Nielsen-Saines K., Baud D. (2023). Potential mechanisms of intrauterine transmission of monkeypox virus. Lancet Microbe.

[61] Cono J., Cragan J.D., Jamieson D.J., Rasmussen S.A. (2006). Prophylaxis and treatment of pregnant women for emerging infections and bioterrorism emergencies. Emerg. Infect. Dis..

[62] Kopanou Taliaka P., Tsantes A.G., Konstantinidi A., Liakou P., Tavoulari E.F., Piovani D., Bonovas S., Iacovidou N., Tsantes A.E., Sokou R. (2023). Monkeypox disease and pregnancy. Where are we today? A review of literature. J. Perinatol..

[63] Mattar R., Neto A.R.B., Luz A.G., Hatanaka A., Zaconeta A., Guazzelli C.A.F., Traina E., Baptista F.S., Osanan G., Duarte G. (2022). Expert Recommendations on Monkeypox (MPX) in Pregnancy, Postpartum and Lactating Women. Rev. Bras. Ginecol. Obstet..

[64] Dashraath P., Nielsen-Saines K., Mattar C., Musso D., Tambyah P., Baud D. (2022). Guidelines for pregnant individuals with monkeypox virus exposure. Lancet.

[65] Dashraath P., Nielsen-Saines K., Rimoin A., Mattar C.N.Z., Panchaud A., Baud D. (2022). Monkeypox in pregnancy: Virology, clinical presentation, and obstetric management. Am. J. Obstet. Gynecol..

